# SuoquanYishen formula improves renal cellular senescence by inhibiting YTHDF1-Rubicon axis to promote autophagy in diabetic kidney disease

**DOI:** 10.3389/fphar.2025.1543277

**Published:** 2025-04-30

**Authors:** Zijie Yan, Lin Zhang, Tianpeng Ma, Yong Yuan, Yu Kang, Shuman Liu, BoCen Chen, Kai Li, Man Xiao, Yiqiang Xie

**Affiliations:** ^1^ College of Traditional Chinese Medicine, Hainan Medical University, Haikou, China; ^2^ Sanya Hospital of Traditional Chinese Medicine, Sanya, Hainan, China; ^3^ Heilongjiang Academy of Traditional Chinese Medicine, Harbin, China; ^4^ Key Laboratory of Tropical Translational Medicine of Ministry of Education, School of Basic Medicine and Life Sciences, Hainan Medical University, Haikou, China; ^5^ Hainan Provincial Key Laboratory for human reproductive medicine and Genetic Research & Hainan Provincial Clinical Research Center for Thalassemia & Key Laboratory of Reproductive Health Diseases Research and Translation, Ministry of Education, Hainan Medical University, Haikou, Hainan, China; ^6^ The First Affiliated Hospital of Hainan Medical University, Haikou, Hainan, China

**Keywords:** diabetic kidney disease, traditional Chinese medicine, SuoquanYishen formula, YTHDF1, Rubicon, autophagy, cellular senescence, m6A modification

## Abstract

SuoquanYishen formula (SQYSF), a traditional Chinese herbal prescription for treating diabetic kidney disease (DKD), has demonstrated clinical efficacy in lowering blood glucose and alleviating renal damage. Emerging evidence implicates cellular senescence as a critical contributor to DKD progression. This study aimed to elucidate the mechanism by which SQYSF improves renal cellular senescence using both *in vivo* (db/db mice) and *in vitro* (high glucose-induced HK-2 cells) DKD models, with interventions involving SQYSF aqueous extract and SQYSF-containing serum. We screened 59 chemical compounds by UHPLC-QTOF-MS and used network pharmacology approach to discover that autophagy and cellular senescence are important pathways for pharmacological treatment of disease. Experimental validation demonstrated that senescence and damage occurred in the kidneys of db/db mice and HK-2 cells under high glucose environment, and SQYSF ameliorated these abnormal changes. Then, we also found that SQYSF enhanced autophagy in renal tissues and cells, whereas co-treatment with the autophagy inhibitor Bafilomycin A1 abolished SQYSF’s anti-senescence effects. Notably, DKD progression was associated with elevated Rubicon expression at mRNA and protein levels, accompanied by increased m6A modification. While SQYSF effectively downregulated Rubicon mRNA and protein expression, it did not influence m6A modification levels. Further investigation identified that SQYSF was able to target to reduce YTHDF1 expression level. Overexpression of YTHDF1 in HK-2 cells increased Rubicon mRNA stability and protein expression, while concurrently reversing SQYSF-induced autophagy enhancement and senescence amelioration. These results suggest that SQYSF exerts its role in ameliorating renal cellular senescence in DKD by targeting to reduce the expression level of YTHDF1, which inhibits the level of Rubicon mRNA and protein translation, and thus promotes autophagy. Our results reveal the active components and mechanisms of SQYSF for the treatment of DKD, which may provide useful information to guide the clinical application of SQYSF as well as the therapeutic pathway for DKD.

## 1 Introduction

Diabetic kidney disease (DKD), a prevalent microvascular complication of diabetes mellitus (DM), constitutes a major risk factor for chronic kidney disease and end-stage renal disease (Rayego-Mateos et al., 2023). According to projected data from the International Diabetes Federation, the number of people with DM worldwide will grow to 643 million and 783 million by 2030 and 2045, respectively. With the global diabetic population increasing, the escalating morbidity and mortality of DKD demand urgent therapeutic solutions (Sun et al., 2022).

Cellular senescence refers to a state of stable cell cycle arrest that can be induced by a variety of factors, including metabolic disorders, DNA damage, epigenetic modification, inflammation and proteotoxic stress (García-Trejo et al., 2024; Di Micco et al., 2021). In recent years, cellular senescence has been shown to play a vital role in the pathologic process of DKD. Senescent cells secrete a number of inflammation-associated cytokines in order to accelerate themselves or to promote neighbouring cells via paracrine signals into an inflammatory state, leading to a continuous accumulation of senescent cells. In short, DM creates an environment that accelerates cellular senescence; conversely, cellular senescence leads to disease progression (Gao et al., 2022). It has been found that the progression of DKD is characterized by accelerated senescence of tubular epithelial cells (Kim MN. et al., 2021), mesangial cells (Cao et al., 2019), podocytes (Fang et al., 2021) and glomerular endothelial cells (Yu et al., 2020). Autophagy, as a mechanism of intracellular content degradation, promotes cell survival by eliminating damaged organelles or harmful substances and thus is a potent regulator of senescence (Chen et al., 2024). Stimulation of autophagy usually delays the aging process (Leidal et al., 2018). Research has shown that inhibition of autophagy activity by Rubicon (an autophagy-associated negative regulator) is considered a hallmark of senescence (Nakamura et al., 2019). In addition, Rubicon has been found to be useful for predicting and detecting the progression of DKD (Watany et al., 2022). N6-methyladenosine (m6A) modification is one of the most common post-transcriptional modifications in organisms (Cheng et al., 2024). There are three main enzymes associated with m6A modification: writers, readers, erasers, which regulate RNA expression levels and are involved in the progression of several renal diseases, including DKD, by adding, recognizing and removing m6A sites (Qi et al., 2023). Although there is evidence that changes in Rubicon expression levels in common metabolic diseases such as Non-alcoholic fatty liver disease (NAFLD) are caused by m6A modification (Peng et al., 2022), it has not been determined whether the underlying mechanisms controlling Rubicon expression levels in DKD are similar to those in NAFLD.

Traditional Chinese medicine (TCM) is widely used as standalone or adjunctive therapies for DKD and have shown good efficacy in clinical practice (Shen et al., 2024; Chung et al., 2023). “Spleen-kidney yang deficiency, Zhenjia accumulation” is an important TCM pathogenetic feature in the development of DKD (Yang R. et al., 2024). SuoquanYishen Formula (SQYSF) is composed of the following seven herbs: Alpiniae oxyphyllae fructus (Yizhiren in Chinese), Linderae Radix (Wuyao in Chinese), poria (Fuling in Chinese), Atractylodis Rhizoma (Cangzhu in Chinese), Salviae Miltiorrhizae Radix et Rhizoma (Danshen in Chinese), Paeoniae Radix Rubra (Chishao in Chinese) and Radix Trichosanthis (Tianhuafen in Chinese), which is effective in “warming the spleen and tonifying the kidneys, clearing up Zhenjia”. Thus, it can effectively target the characteristics of TCM pathogenesis of DKD. SQYSF has been in clinical use for more than 10 years, with proven efficacy in DKD, and has been granted a patent for its invention (Patent No: ZL 201610423677.5). Our previous study reported that SQYSF was effective in improving clinical symptoms, decreasing urinary protein, improving renal function, decreasing serum inflammatory factors, and reducing renal microinflammatory status in patients with DKD (Yan et al, 2024). The formula’s monarch medicine-Alpiniae oxyphyllae fructus attenuates age-related phenotypes and prolongs lifespan in *Caenorhabditis elegans* (Xiao et al., 2022), and Alpiniae oxyphyllae fructus also showed good potential to ameliorate renal cell senescence in DKD (Yan et al., 2024a). Some researchers have also found that treatments of “warming the spleen and tonifying the kidneys or clearing up Zhenjia” are closely related to the improvement of cellular senescence or aging (Wang L. et al., 2023; Wen et al., 2022). Therefore, in terms of TCM theory and modern findings, SQSYF has the potential to ameliorate cellular senescence, but its specific mechanism remains to be probed.

This study investigates whether SQYSF alleviates renal cellular senescence in DKD by targeting to reduce YTHDF1, thus inhibiting Rubicon mRNA and protein translation to promote autophagy. Our results provide a reliable experimental basis for the exploration of innovative drugs for treating DKD, detailed mechanistic studies and clinical trials, and also provide a theoretical basis for the internationalisation and modernisation of TCM.

## 2 Material and methods

### 2.1 Chemicals and reagents

Actinomycin D, Canagliflozin (HY-17559; HY-10451, MCE, Shanghai, China); Bafilomycin A1 (Baf A1) (T6740, TargetMol, Shanghai, China); 45% D-(+)-dextrose solution (G8769, Sigma, Shanghai, China); Creatinine (Cr) Assay kit, blood urea nitrogen (BUN) assay kit (C011-2-1; C013-2-1, Nanjing Jiancheng, Nanjing, China); Mouse Urinary Microalbumin (mAlb) ELISA Kit (F2702-A, FANKEW, Shanghai, China); Eastep^®^ Super Total RNA Extraction Kit (LS1040, Promega, Shanghai, China); Hifair^®^ Ⅲ 1st Strand cDNA Synthesis SuperMix for qPCR (gDNA digester plus), Hieff^®^ qPCR SYBR Green Master Mix (Low Rox Plus), Peroxidase-Conjugated Rabbit Anti-Goat IgG (H + L) (11141ES; 11202ES; 33707ES, 34850ES60, Yeasen, Shanghai, China); Senescence β-Galactosidase Staining Kit (C0602, Beyotime, Shanghai, China); RIPA lysate, BCA Protein Quantification Kit, Omni-Easy™ One-Step PAGE Gel Rapid Preparation Kit, Omni-Easy™ Instant Protein Sampling Buffer (PC101; ZJ102; PG210-PG214; LT101, Epizyme, Shanghai, China); MeRIP kit (Bes5203, Bersin, Guangzhou, China); Glycogen PAS staining solution, Hematoxylin and Eosin (HE) Staining Kit (R20526; R32933, Yuanye, Shanghai, China). Information about the antibodies is showed in Supplementary Table S1.

### 2.2 Animals

35–48 days old SD rats, 4 weeks old db/m and db/db mice were purchased by Gempharmatech (Nanjing, China). All animals were housed in Hainan Yunliang Biotechnology Co. (laboratory environment: temperature 22°C ± 2°C, humidity 50% ± 5%, 12-h light/dark cycle). Animal studies were approved by the Animal Care and Ethics Committee of Hainan Medical University, no. HYLL-2021-389.

### 2.3 Preparation of SQSYF decoction

Herbs were purchased from the First Affiliated Hospital of Hainan Medical University. Firstly, 80 g of SQSYF (10 g Yizhiren, 10 g Wuyao, 15 g Fuling, 10 g Cangzhu, 15 g Danshen, 10 g Chishao, 10 g Tianhuafen) was soaked in 8 times the volume of sterile water for 60 min and then decocted for 60 min. The above process was repeated twice and then the two decoctions were mixed and concentrated by rotary evaporator to 2 g/mL. The above extracts were subjected to UHPLC-QTOF-MS for composition analysis and quality control based on previously established methodology (Yan et al., 2024a).

### 2.4 Network pharmacology analysis and molecular docking

The compounds obtained from mass spectrometry were screened by TCMSP (https://www.tcmsp-e.com/) using the criteria of oral bioavailability (OB) > 30% and drug-likeness (DL) > 0.18 (Ru et al., 2014). Access to herbs-related targets through Pubchem (https://pubchem.ncbi.nlm.nih.gov/) (Kim S. et al., 2021) and Swisstarget Prediction (http://www.swisstargetprediction.ch/) (Daina et al., 2019) and DKD-related targets through OMIM (http://www.omim.org) (Amberger et al., 2015) and genecards (https://www.genecards.org/) databases (Stelzer et al., 2016). Common targets for drugs and diseases are obtained through Venny 2.1.0 (https://bioinfogp.cnb.csic.es/tools/venny/index.html). After gene ontology (GO) and kyoto encyclopedia of genes and genomes (KEGG) enrichment analysis through the DAVID database (https://david.ncifcrf.gov/) (Sherman et al., 2022), visualization was performed using bioinformatics (https://www.bioinformatics.com.cn/). The crystal structure of YTHDF1 (PDB ID:8BS4) was obtained from the PDB database (http://www.rcsb.org/pdb/) (Burley et al., 2023). Molecular docking was performed after processing of proteins and small molecule compounds using PyMOL, AutoDock Vina 1.1.2 and Open Bable GUL. Finally, the ligand-protein conformation with the lowest binding energy was visualized using PyMOL software.

### 2.5 Animal experiments

After 2 weeks of acclimatization, db/db mice were randomly assigned to Model group, low-dose SQYSF group (LSQYSF, 5 mg/g/day), medium-dose SQYSF group (MSQYSF, 10 mg/g/day), high-dose SQYSF group (HSQYSF, 20 mg/g/day), Canagliflozin group (12.5 mg/kg/day Canagliflozin). db/m mice served as Normal group. The random blood glucose (GLU) and body weight were measured every 2 weeks. After 8 weeks of drug intervention, 24-hour urine volume was collected and blood samples were taken. The blood samples were then centrifuged at 3,000 rpm/15 min/4°C to collect serum samples. Urine microalbumin (mAlb), serum creatinine (Scr) and blood urea nitrogen (BUN) levels were measured using appropriate kits.

### 2.6 Histopathological and immunohistochemistry (IHC) examination

The kidneys were fixed with 4% paraformaldehyde, embedded in paraffin, and sectioned. The prepared kidney sections were stained with hematoxylin and eosin (HE) and Passon stainings according to the kit protocols. In addition, kidney sections were stained for IHC using antibodies against YTHDF1, P16, P21, and P53. Finally, the positive area was quantified using ImageJ software.

### 2.7 Cell experiments

#### 2.7.1 Preparation of SQYSF-containing serum

SD rats, acclimatized for 1 week, were randomly divided into two groups: the SQYSF group and the Control group. The rats in the SQYSF group were gavaged once a day at a dose of 72 g/kg/day for seven consecutive days. The rats in the control group were given an equal amount of saline. One hour after the last administration, blood was obtained under anesthesia. The blood was then centrifuged at 3,000 rmp/min for 10 min at 4°C to collect the SQYSF-containing serum and blank serum. The serum is heat-inactivated at 56°C for 30 min. The prepared medium was filtered through a 0.22 μm filter prior to administration of the cellular intervention.

#### 2.7.2 Cell culture and lentiviral transfection

HK-2 cells (purchased from Pricella, Wuhan, China) were cultured in MEM medium with 10% fetal bovine serum or rat serum and 1% penicillin/streptomycin in an incubator at 37.2°C, 80% humidity, 5% CO_2_. To construct a stable YTHDF1-overexpressing cell line, a viral vector with the name pcSLenti-CMV-YTHDF1-3×FLAG-PGK-Puro-WPRE3 was purchased from obiosh (Shanghai, China), along with a negative control with the name pcSLenti-CMV-MCS-3×FLAG-PGK-Puro-WPRE3. The cells were infected for 12 h in 6-well plates using polybrene-plus and a certain amount of virus. Following infection, puromycin was added to the medium for long-term culture to select stably overexpressing cell lines.

#### 2.7.3 CCK8

Exponentially growing HK-2 cells were inoculated into 96-well plates and cultured for 24 h. The cells were exposed to high glucose intervention for 72 h and drug treatment for 24 h. Then, the optical density value of each well was determined at 450 nm using the CCK8 assay kit.

#### 2.7.4 Cellular thermal shift assay (CETSA)

HK-2 cells were cultured for 2 days to about 70% density, followed by a 24-h intervention with either SQYSF-containing serum or blank serum. After trypsin digestion, the cell number was counted using a cell counter, and an equal number of cells were collected, centrifuged and washed once with PBS, followed by collection of cell precipitates. Two groups of cells were resuspended by adding 600 μL PBS and the liquid was equally divided into six PCR tubes. The PCR tubes were placed in a scilogex TC1000-g PCR instrument and heated for 3 min, and the temperatures of the six tubes were set to 45, 48, 51, 54, 57, and 60°C. The processed cells were repeatedly frozen and thawed by liquid nitrogen for three times, and then denatured with protein sampling buffer for 10 min at 100°C. Finally, the protein samples were used for subsequent Western blot detection.

#### 2.7.5 RNA stability assay

HK-2 cells were divided into three groups: Normal, OE-NC, OE-YTHDF1. Stable cell lines overexpressing YTHDF1 and normal cells were counted and inoculated in 6-well plates according to the same number. After 48 h of incubation, HK-2 cells were processed with 5 μg/mL actinomycin D for 6 h, 4 h, 2 h, 1 h, and 0 h, respectively. Finally, total RNA was extracted from the cell samples and reverse transcribed to cDNA using the appropriate kits for subsequent RT-qPCR analysis.

#### 2.7.6 Transmission electron microscopy

The trypsin-digested cell samples were collected and fixed with electron microscope fixative for 30 min at room temperature away from light, and then the samples were transferred to 4°C for storage. Subsequently, the samples were encapsulated and sliced. Finally, images of autophagosomes and autolysosome were taken using transmission electron microscope (Hitachi, Japan).

### 2.8 SA-β-gal staining

The medium in the 6-well plate was aspirated and washed three times with PBS for 3 min for cell samples and the prepared frozen sections were rewarmed for 10 min at room temperature for kidney samples. β-Galactosidase staining fixative was then added to the samples for 15 min. Subsequently, the staining solution was added to both the 6-well plates and kidney sections, which were immediately sealed. After incubation for 12 or 24 h at 37°C in a CO_2_-free incubator, the samples were observed and photographed. The blue staining was considered as the aggregation area of senescent cells.

### 2.9 MeRIP-qPCR

Total RNA was extracted by grinding an appropriate amount of kidney tissue using a grinder, and the RNA is then fragmented and immunoprecipitated using the MeRIP m6A kit (BersinBio, Guangzhou). Protein A/G magnetic beads were prepared and conjugated to IgG or m6A antibodies, followed by a second round of RNA extraction. Subsequently, the expression level of Rubicon mRNA in each sample was determined by RT-qPCR and normalized by Input value (without immunoprecipitation).

### 2.10 RT-qPCR

Total RNA was extracted from kidney tissues or cells, and RNA purity and concentration were assessed using a Nanodrop 2000 spectrophotometer to ensure they were within the acceptable range. The RNA was then reverse transcribed into cDNA. Primers (Supplementary Table S1) synthesized from Tsingke (Beijing, China), cDNA and Mix were mixed and analyzed using a q225 fluorescence quantitative PCR instrument (KUBO, Beijing, China). The mRNA expression levels of target genes were normalized to β-actin and calculated using the 2^−ΔΔCt^ method.

### 2.11 Western blot

Appropriate amounts of cells or kidneys were taken and lysed with the addition of RIPA and PMSF to obtain total proteins. After determining the protein concentration with BCA protein quantification kit, the protein concentration of each group of samples was adjusted to be the same. A 0.25-fold volume of loading buffer was added and the samples were denatured in a metal bath at 100°C/10 min. The prepared protein samples are electrophoresed and transferred to PVDF membrane. Membrane blocking, primary antibody incubation, secondary antibody incubation and visualization are then performed sequentially. The relative expression levels of the proteins were analyzed by ImageJ using β-actin as reference.

### 2.12 Statistical analysis

All Experimental data are presented as mean ± standard deviation at least three independent experiments. One-way analysis of variance (ANOVA) was used for multiple comparisons and Tukey method was used to compare any two sets of data. Graphpad Prism (Version 9.4.1) was used to statistical analysis and diagram generation. *P* value of less than 0.05 was considered to have a statistically significant difference.

## 3 Results

### 3.1 UHPLC-QTOF-MS analysis of SQYSF related components

**FIGURE 1:**
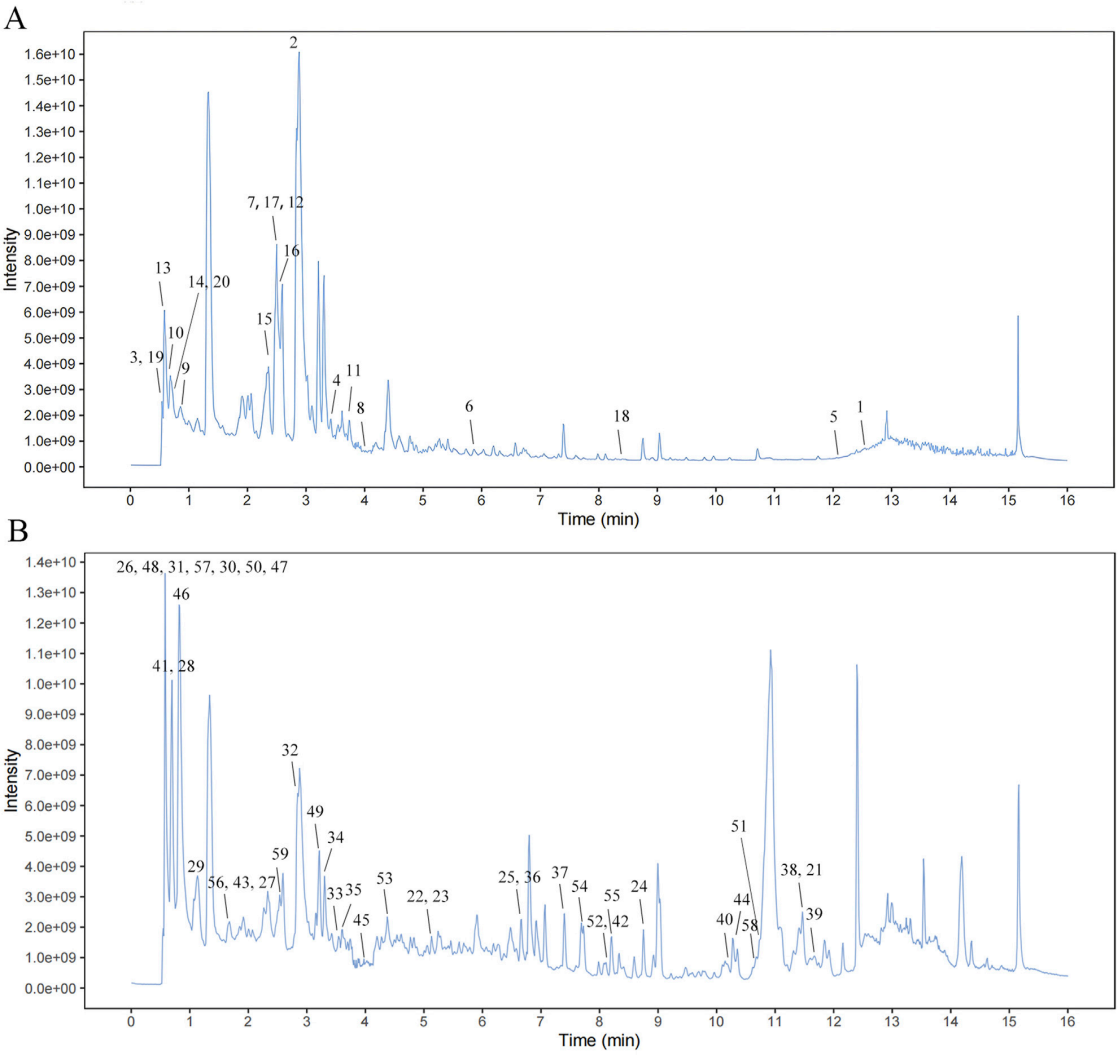
Mass spectrum chromatograms of SQYSF. Negative mode **(A)** and Positive mode **(B)**.

UHPLC-QTOF-MS was used to identify the representative chemical components of SQYSF (Figures 1A,B). A total of 55 compounds (four compounds were detected in both positive and negative ions) and their sources were identified (Supplementary Table S2).

### 3.2 Network pharmacology analysis

**FIGURE 2:**
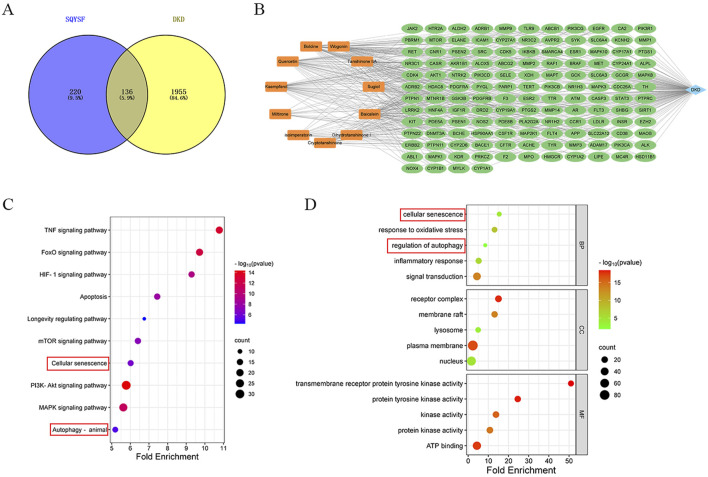
The result of network pharmacology analysis. Venn diagram of SQYSF and DKD targets **(A)**; compounds-therapeutic targets-disease network **(B)**. The main pathways and biological processes in KEGG **(C)** and GO **(D)** enrichment analysis.

Eleven compounds (Boldine, Quercetin, Wogonin, Cryptotanshinone, Sugiol, Kaempferol, Tanshinone IIA, Miltirone, isoimperatorin, Dihydrotanshinone I, Baicalein) were screened for network pharmacology analysis using OB>30%, DL > 0.18 as criteria. A total of 356 drug-related targets were obtained from SwissTargetPrediction database, and 2091 disease-related targets were obtained from GeneCards and OMIM databases. Detailed information is provided in Supplementary Table S3. By intersecting the targets of SQSYF with those related to DKD, 136 potential therapeutic targets were identified (Figure 2A). A “compounds-targets-disease” network was subsequently constructed using Cytoscape 3.9.1 software (Figure 2B). The 136 common targets were further analyzed using the DAVID database for GO and KEGG analysis, with P < 0.05 set as the threshold for significant biological function. Further details are available in Supplementary Table S4. KEGG pathway enrichment analysis yielded a total of 149 signaling pathways associated with the treatment of DKD by SQYSF, including cellular senescence, autophagy, mTOR signaling pathway, longevity regulating pathway, TNF signaling pathway (Figure 2C). The results of GO enrichment analysis showed that 481 biological processes (BP), 77 cellular components (CC), and 109 molecular function (MF) were identified. In the GO_BP category, the terms were mainly involved signal transduction, response to oxidative stress, inflammatory response, and other functions. In the GO_BP category, the terms were mainly involved signal transduction, response to oxidative stress, inflammatory response, cellular senescence, regulation of autophagy. In the GO_CC category, the terms mainly included receptor complex, lysosome, nucleus (Figure 2D). These results suggest that cellular senescence and autophagy may play crucial roles in the therapeutic effects of SQYSF on DKD.

### 3.3 SQYSF lowerd blood glucose and improved kidney function and pathologic tissue damage in DKD mice

**FIGURE 3:**
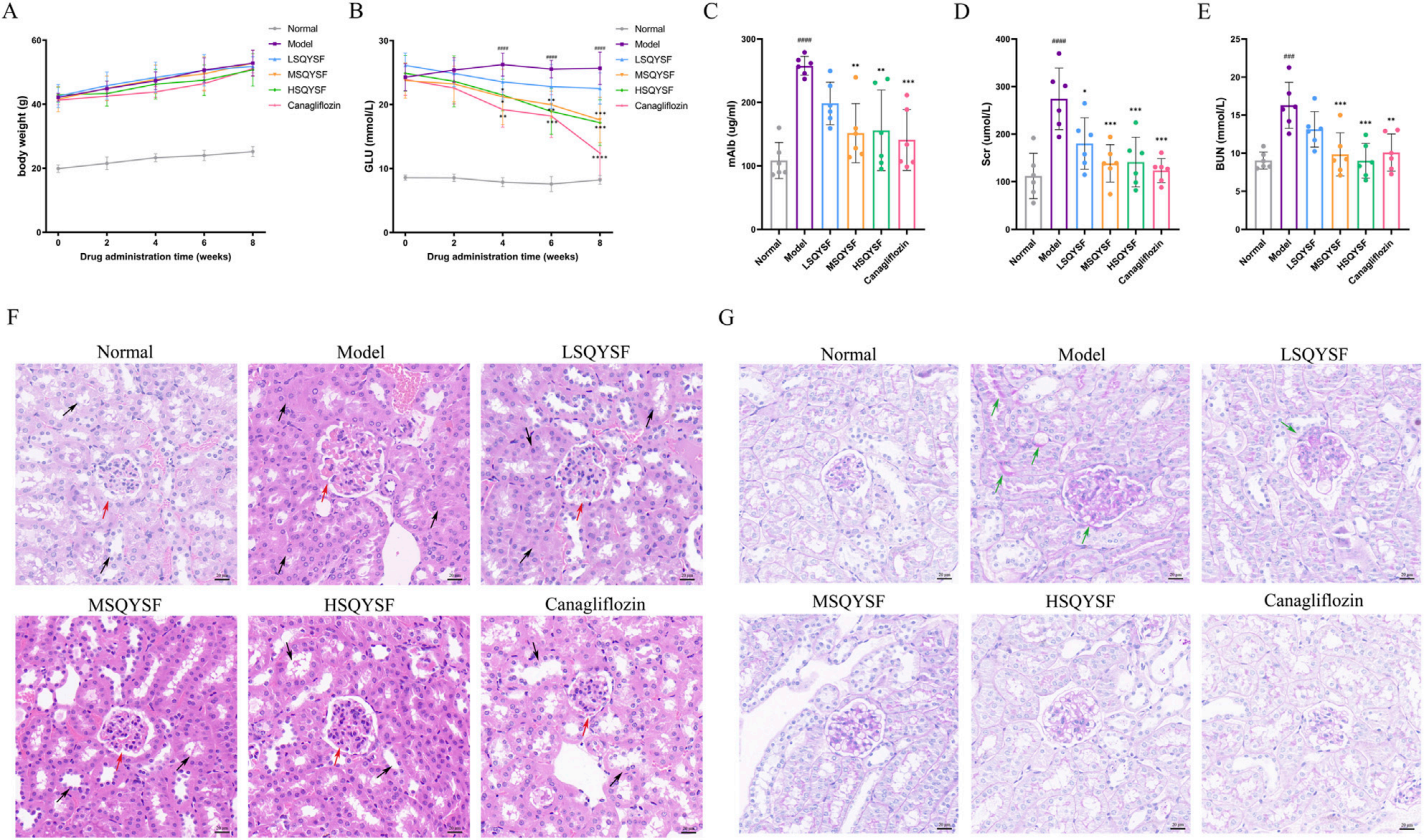
Efficacy of SQYSF on blood glucose, kidney function and pathological tissue in DKD mice. Body weight at 0, 2, 4, 6, 8th weeks of administration **(A)**; GLU levels at 0, 2, 4, 6, 8th weeks of administration **(B)**; mAlb **(C)**, Scr **(D)**, and BUN **(E)** levels after 8 weeks of administration. ###*P* < 0.001, ####*P* < 0.0001 compared with the normal group, *P < 0.05, **P < 0.01, ***P < 0.001 and ****P < 0.0001 compared with the model group. Histopathological changes in kidney tissues evaluated by HE staining **(F)** and Passon staining **(G)**. The original magnification was ×40 and the scale bar represents 20 μm (The red arrow points towards the glomerulus, the black arrow point towards the renal tubule, the green arrows point to areas of abnormally increased glycogen deposition).

To evaluate the effects of SQYSF on lowering blood glucose and protecting kidney, DKD mice were treated with canagliflozin and different doses of SQYSF for 8 weeks. During the experimental period, the GLU levels in the model group were significantly higher than those in the normal group. Although SQYSF did not have a significant effect on body weight, GLU levels gradually decreased after the fourth week of treatment compared with untreated db/db mice (Figures 3A,B). DKD is clinically characterized by an increase in mAlb excretion and impaired renal function, including elevated Scr and BUN levels. Compared with normal mice, the model group exhibited significantly higher mAlb, Scr, and BUN levels. After 8 weeks of SQYSF treatment, these levels were notably reduced (Figures 3C–E). In order to further investigate the recovery of renal pathological tissue damage, HE staining and Passon staining were performed. HE staining showed that the glomeruli and tubules in the normal group were structurally intact and morphologically clear, specifically, the glomerular capsule lumen was clearly visible, there was no obvious hyperplasia of the basement membrane and stroma, and the tubules were arranged in a regular pattern. In contrast, the model group showed increased glomerular swelling, thickening of the basement membrane, swelling of tubular epithelial cells, and narrowing of the tubular lumen. After drug intervention, the renal pathological changes in each administration group were reduced to different degrees (Figure 3F). Passon staining showed that there was more glycogen deposition in glomeruli and tubules in the model group, and the administration of SQSYF or canagliflozin reduced glycogen deposition in renal tissues (Figure 3G). Furthermore, we observed that the medium- and high-dose SQYSF treatments were similarly effective as canagliflozin in improving renal function and mitigating pathological tissue damage, with both doses proving more effective than the low-dose SQYSF treatment.

### 3.4 SQYSF improved cellular senescence and restored autophagy function in kidney of DKD mice

**FIGURE 4:**
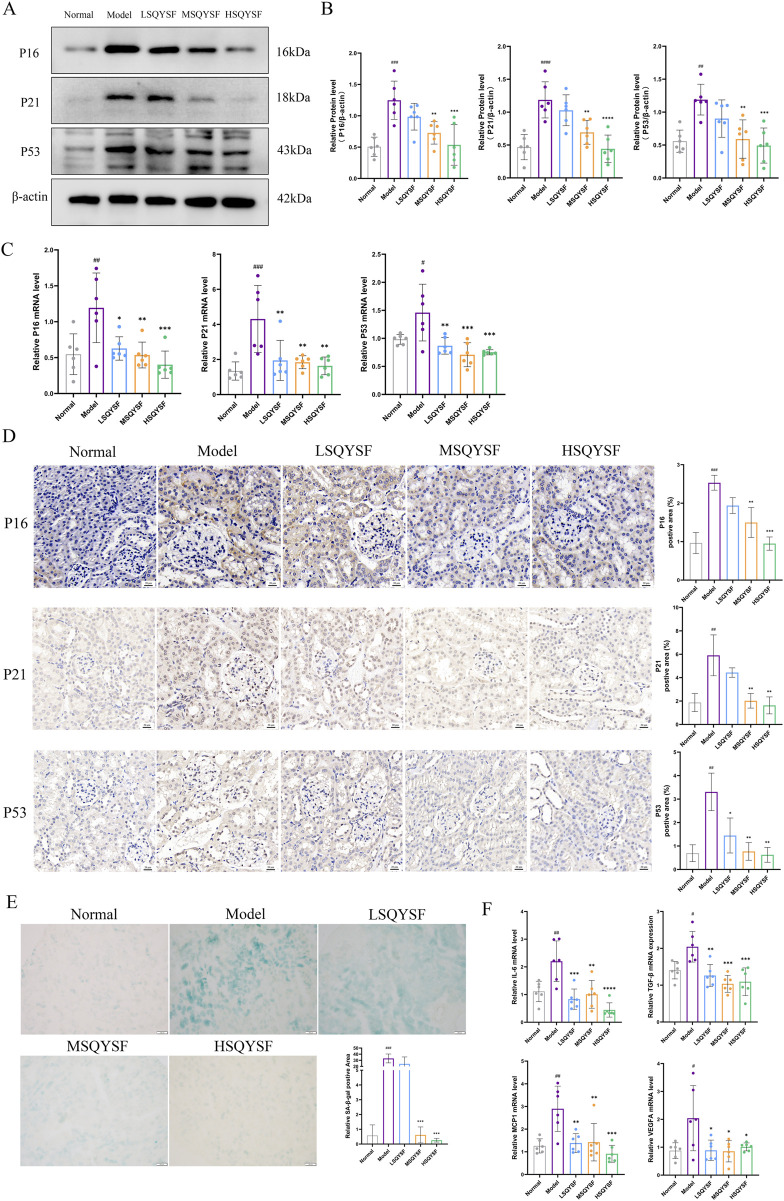
SQYSF improved cellular senescence in kidney of DKD mice. Original bands of P16, P21, P53 proteins **(A)** and quantitative analysis **(B)**. P16, P21, P53 mRNA expression levels **(C)**. Immunohistochemical staining images of P16, P21, P53 **(D)**, the scale bar represents 20 μm; Positive area of SA-β-Gal staining in kidney **(E)**, the scale bar represents 100 μm; IL-6, TGF-β, MCP1, VEGFA mRNA expression levels **(F)**; #*P* < 0.05, ##*P* < 0.01, ###*P* < 0.001, ####*P* < 0.0001 compared with the normal group, *P < 0.05, **P < 0.01, ***P < 0.001 and ****P < 0.0001 compared with the model group.

A substantial body of evidence suggests that extensive renal cellular senescence can occur in diabetic environments (Gao et al., 2022). In this study, We first investigated whether SQYSF could improve renal cellular senescence in DKD mice. Cellular senescence, characterized by permanent cell cycle arrest, is primarily initiated through two key signaling pathways: p53-p21 and p16-RB, which together maintain the growth arrest phenotype. Therefore, the expression levels of cell cycle inhibitors-P16, P21, and P53 are often used as markers of cellular senescence. Compared with the normal group, P16, P21, P53 mRNA and protein expression levels were significantly increased in the model group. However, these levels were decreased after the intervention of SQYSF (Figures 4A–C). Similar results were obtained by IHC analysis (Figure 4D). SA-β-gal activity is upregulated when cellular senescence occurs. Therefore, the expression level of β-galactosidase activity can be measured in senescent cells or tissues to reflect the state of cellular senescence (Childs et al., 2017). Compared with the normal group, the positive area of SA-β-Gal staining in the kidney tissues of mice in the model group was significantly elevated, whereas it was decreased after SQYSF intervention (Figure 4E). Senescent cells can secrete a variety of cytokines collectively known as senescence-associated secretory phenotype (SASP) including IL-6, TGF-β, MCP-1, VEGFA, and many other cytokines, which can affect the local microenvironment and lead to chronic inflammation, thereby enhancing the senescent phenotype of their own and surrounding cells (Birch and Gil, 2020). The results showed that the expression levels of IL-6, TGF-β, MCP1, VEGFA mRNA were elevated in the renal tissues of db/db mice in the model group compared with the normal group by RT-qPCR. Notably, SQYSF intervention resulted in a marked reduction in the expression of IL-6, TGF-β, MCP1, VEGFA mRNA (Figure 4F).

**FIGURE 5:**
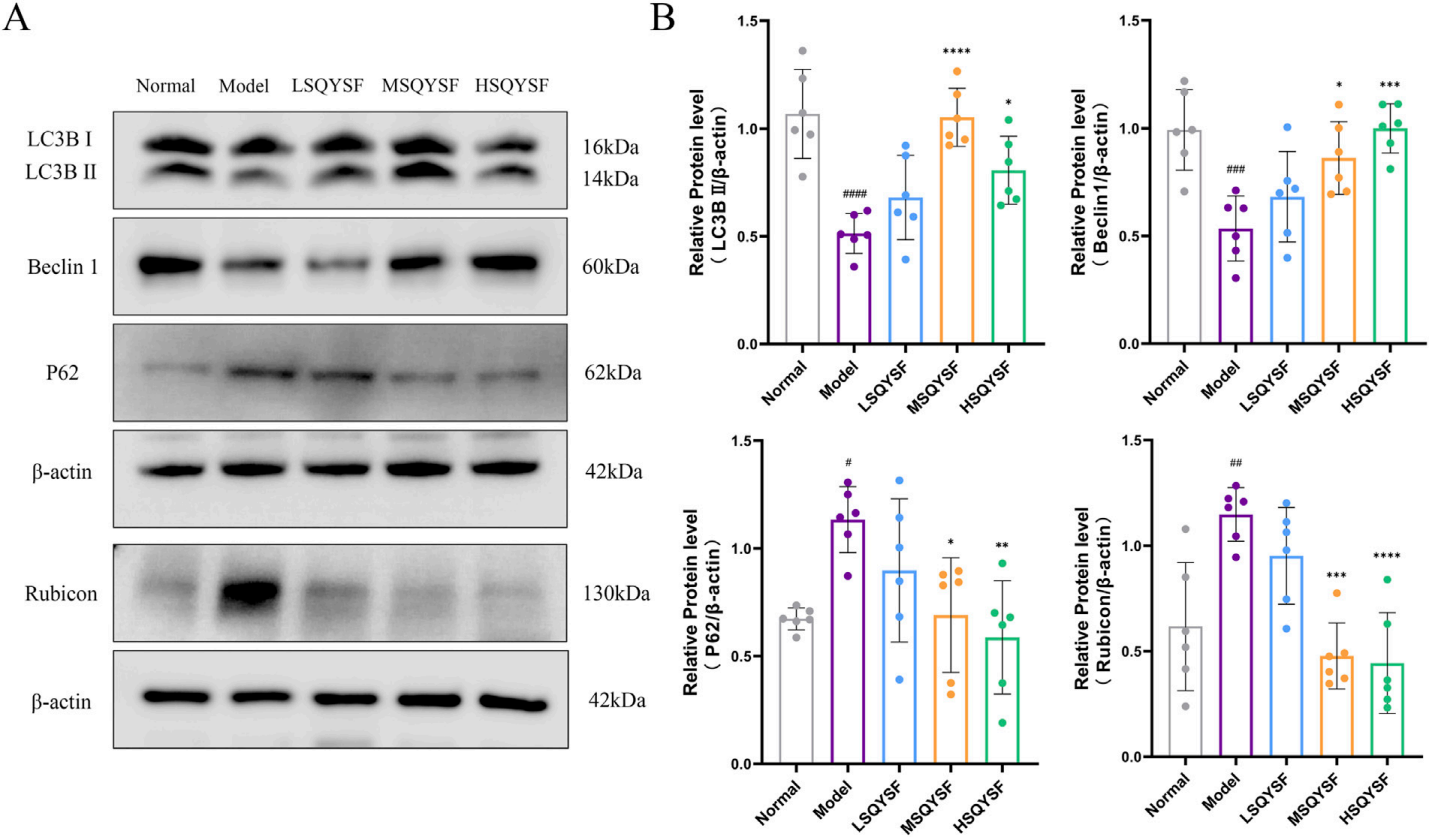
SQYSF restored autophagy function in kidney of DKD mice. Original bands of LC3B, P62, Beclin1, Rubicon proteins **(A)** and quantitative analysis **(B)**. #*P* < 0.05, ##*P* < 0.01, ###*P* < 0.001, ####*P* < 0.0001 compared with the normal group, *P < 0.05, **P < 0.01, ***P < 0.001 and ****P < 0.0001 compared with the model group.

There is increasing evidence that autophagy is inhibited during the progression of DKD. Restoration of autophagy function can reduce the accumulation of senescent cells under certain pathological conditions (Tang et al., 2020). Consequently, we investigated whether SQYSF could restore autophagic function. Our results demonstrated that, compared with the normal group, the autophagy levels were reduced in the model group, as evidenced by the reduced expression levels of Beclin1 and LC3B II proteins and the increased expression level of P62 and Rubicon proteins. However, following SQYSF intervention at medium and high doses, autophagy levels were significantly increased, and the expression of these key proteins returned closer to normal levels (Figures 5A,B).

### 3.5 SQYSF-containing serum ameliorates HK-2 cell senescence under high glucose condition by promoting autophagy

**FIGURE 6:**
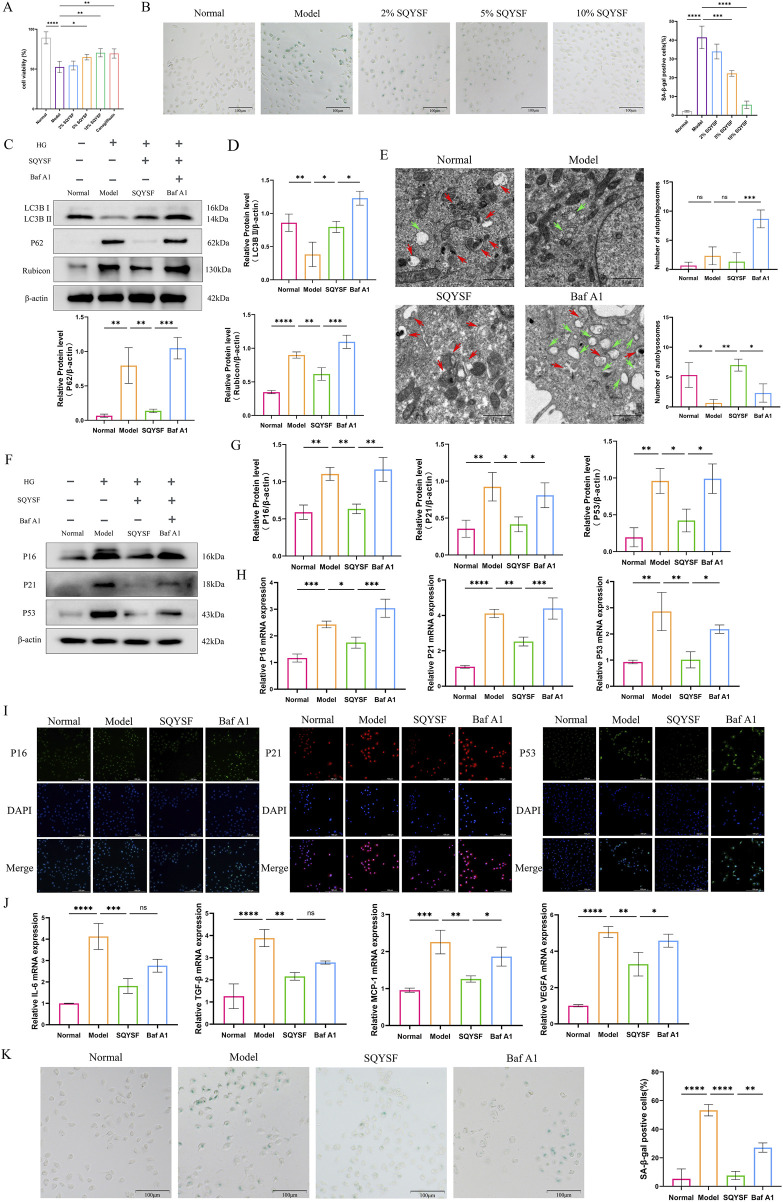
SQYSF-containing serum ameliorates HK-2 cell senescence under high glucose condition by promoting autophagy. Effects of different doses of SQYSF-containing serum on HK-2 cell viability **(A)** and the number of SA-β-gal staining positive cells **(B)**; Original bands **(C)** and quantitative analysis of LC3B Ⅱ, Rubicon, and P62 proteins **(D)**; The number of autophagosome (green arrows) and autolysosome (red arrows) **(E)**; Original bands **(F)** and quantitative analysis **(G)** of P16, P21, P53 proteins; P16, P21, P53 mRNA expression levels **(H)**; Fluorescence intensity of P16, P21, P53 **(I)**; IL-6, TGF-β, MCP1, VEGFA mRNA expression levels **(J)**; SA-β-gal staining positive cells **(K)**. All data are expressed as means ± SD (n = 3). *P < 0.05, **P < 0.01, ***P < 0.001 and ****P < 0.0001.

We used a high glucose (60 mM) intervention in HK-2 cells for 72 h as an *in vitro* model of DKD, based on our previous studies (Yan et al., 2024b). CCK8 and SA-β-gal staining revealed that 10% SQYSF-containing serum was the optimal concentration for restoring cell viability and ameliorating cellular senescence (Figures 6A,B), and thus this dose was chosen for the subsequent experiments. To investigate whether SQYSF ameliorates HK-2 cell senescence by promoting autophagy, we performed a reverse verification *in vitro* by used Baf A1 (5 nM) (which inhibits the fusion of autophagosome and lysosome) to block the autophagy-promoting effect of SQYSF, and at the same time to observe whether the SQYSF-containing serum still possesses the therapeutic efficacy of ameliorating HK-2 cell senescence. Western blot showed that high glucose intervention inhibited autophagy in HK-2 cells compared with the normal group, as evidenced by a decrease in the expression levels of LC3B II protein and an increase in the expression level of P62, Rubicon protein; whereas after intervention with SQYSF-containing serum, the expression levels of LC3B II protein were elevated and the level of P62, Rubicon proteins were decreased, which indicated that SQYSF can promote autophagy in HK-2 cells under high glucose condition. In addition, the addition of Baf A1 to the drug treatment showed an increase in the expression level of P62, Rubicon proteins compared to the SQYSF group, but to our surprise, the expression level of LC3B II did not decrease, but rather increased (Figures 6C,D), which may be attributed to the fact that the Baf A1 led to the blockage of the autophagic late pathway, which suppressed the fusion of autophagosomes with lysosomes, and which led to the abnormal accumulation of autophagosomes. Subsequently, we observed the number of autophagosomes and autolysosome by transmission electron microscopy. Compared with the normal group, the number of autophagosomes and autolysosome was reduced in the model group, while the number of autophagosomes and autolysosome was increased by the drug intervention, and a significant increase in the number of autophagosomes was found after the intervention of Baf A1, whereas the number of autolysosome was significantly reduced (Figure 6E), which further corroborated the results of Western blot. In conclusion, these results indicate that the number of autophagosome and autolysosome is significantly reduced in diabetic environment, and the SQYSF-containing serum can not only improve the formation of autophagosomes, but also promote the formation of autolysosome to activate autophagy, and this effect can be blocked by Baf A1. Then, it was found that SQYSF-containing serum was able to improve the senescence of HK-2 cells under high glucose stress by RT-qPCR, immunofluorescence and Western blot, which was manifested by decreasing the expression levels of P16, P21 and P53 (Figures 6F–I), and decreasing the expression levels of IL-6, TGF-β, MCP-1, and VEGFA mRNA (Figure 6J), and the effect of this change could also be reversed by Baf A1. In addition, Baf A1 also reversed the efficacy of SQYSF-containing serum in reducing SA-β-gal activity (Figure 6K).

### 3.6 SQYSF targets to inhibit YTHDF1 to regulate rubicon mRNA stability and protein level

**FIGURE 7:**
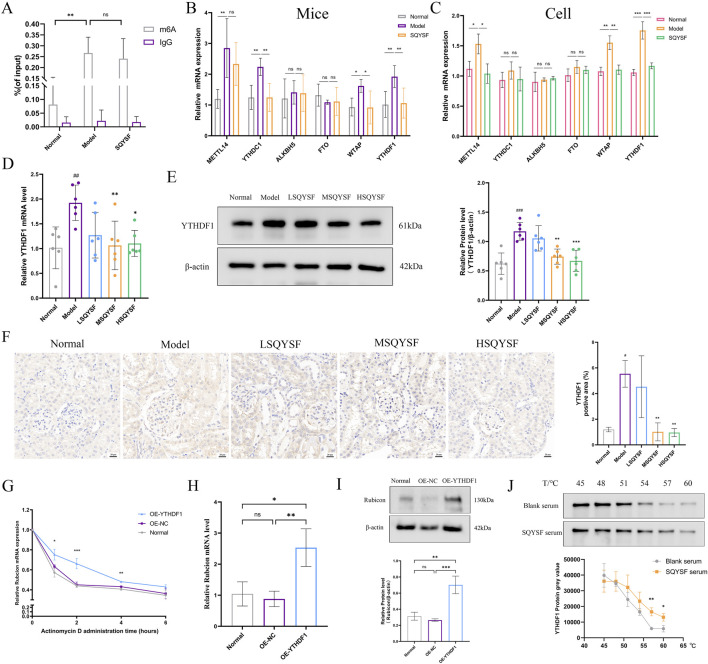
SQYSF targets to inhibit YTHDF1 to regulate Rubicon mRNA stability and protein level. Rubicon m6A modification levels in the kidneys of DKD mice (n = 5) **(A)**; mRNA expression levels of genes related to m6A methylation in mouse kidneys (n = 5) **(B)** and HK-2 cells (n = 3) **(C)**; The effect of different doses of SQYSF on the expression level of YTHDF1 in the kidneys of DKD mice were detected by RT-qPCR **(D)**, Western blot **(E)** and immunohistochemistry **(F)**; Effect of overexpression of YTHDF1 on Rubicon mRNA stability **(G)**, mRNA **(H)** and protein **(I)** expression levels in HK-2 cells; Effects of SQYSF-containing serum on the heat stability of YTHDF1 protein in HK-2 cells **(J)**; #*P* < 0.05, ##*P* < 0.01, ###*P* < 0.001 compared with the normal group and *P < 0.05, **P < 0.01, ***P < 0.001 compared with the model group for Figures 7D–F.

Increased expression levels of Rubicon, a known negative regulator of autophagy, have been proposed as a potential biomarker for DKD and can aid in monitoring disease progression (Watany et al., 2022). It has been reported in the literature that Rubicon may undergo m6A modification (Peng et al., 2022), which could lead to alterations in its mRNA and protein expression. To explore this, we firstly predicted the m6A loci in Rubicon by SRAMP database, and the results showed that there were 52 potential m6A loci in Rubicon, including 11 ultra-high-confidence predicted loci and 13 high-confidence predicted loci (Supplementary Figure S1). Then MeRIP-qPCR revealed that the m6A modification level of Rubcion mRNA was significantly increased in the kidney of DKD mice compared with normal mice. However, it was very interesting that there was no significant change in the m6A modification level on Rubcion mRNA after the intervention of SQYSF compared with the model group (Figure 7A). Subsequently, we examined the changes in the expression of molecules associated with m6A modification by RT-qPCR, and the results showed that SQYSF was able to significantly reduce the expression levels of YTHDC1, WTAP, and YTHDF1 mRNAs in renal tissues of DKD mice (Figure 7B), and that SQYSF-containing serum was able to significantly reduce the expression levels of METTL14, WTAP, and YTHDF1 mRNA in high glucose-induced HK-2 cells (Figure 7C). Therefore, we guessed that the expression change of reader molecule-YTHDF1 is more likely to be an important molecule for SQYSF to regulate the expression level of Rubicon. Then, RT-qPCR, Western blot and immunohistochemistry also showed that SQYSF reduced YTHDF1 mRNA and protein expression levels in kidneys of DKD mice, and the expression levels were changed in a dose-dependent manner (Figures 7D–F). Therefore, the targeted regulation of YTHDF1 by SQYSF may represent a crucial mechanism in the treatment of DKD. In the following experiments, we first constructed a YTHDF1-overexpression cell line and detected the overexpression efficiency of YTHDF1 by RT-qPCR, Western blot, and immunofluorescence (Supplementary Figure S2). In addition, to further investigate whether YTHDF1 could promote Rubicon mRNA stability and protein translation, we performed the following experiments. The results of RNA stability assay by adding actinomycin D showed that overexpression of YTHDF1 significantly reduced the degradation level of Rubicon mRNA (Figure 7G); RT-qPCR and Western blot results showed that overexpression of YTHDF1 significantly increased the levels of Rubicon mRNA and protein expression (Figures 7H,I). We then verified the docking ability between YTHDF1 and 11 chemical components of SQYSF by molecular docking simulation. The results showed that the docking binding energies were less than −1.2 kcal/mol (Supplementary Figure S3), which indicated that the compounds had a good binding ability to the target. To further validate that SQYSF-containing serum can effectively bind to YTHDF1, we performed CETSA in HK-2 cells. The results showed that, upon increasing the temperature, the expression level of YTHDF1 protein in the SQYSF serum group was higher than that in the blank serum group (Figure 7J). This indicates that the active ingredient of SQYSF improves the thermal stability of YTHDF1 protein, makes YTHDF1 more tolerant to the heat treatment, and has the possibility to bind YTHDF1.

### 3.7 SQYSF promotes autophagy and ameliorates cellular senescence in HK-2 cells by inhibiting the YTHDF1-Rubicon axis

**FIGURE 8:**
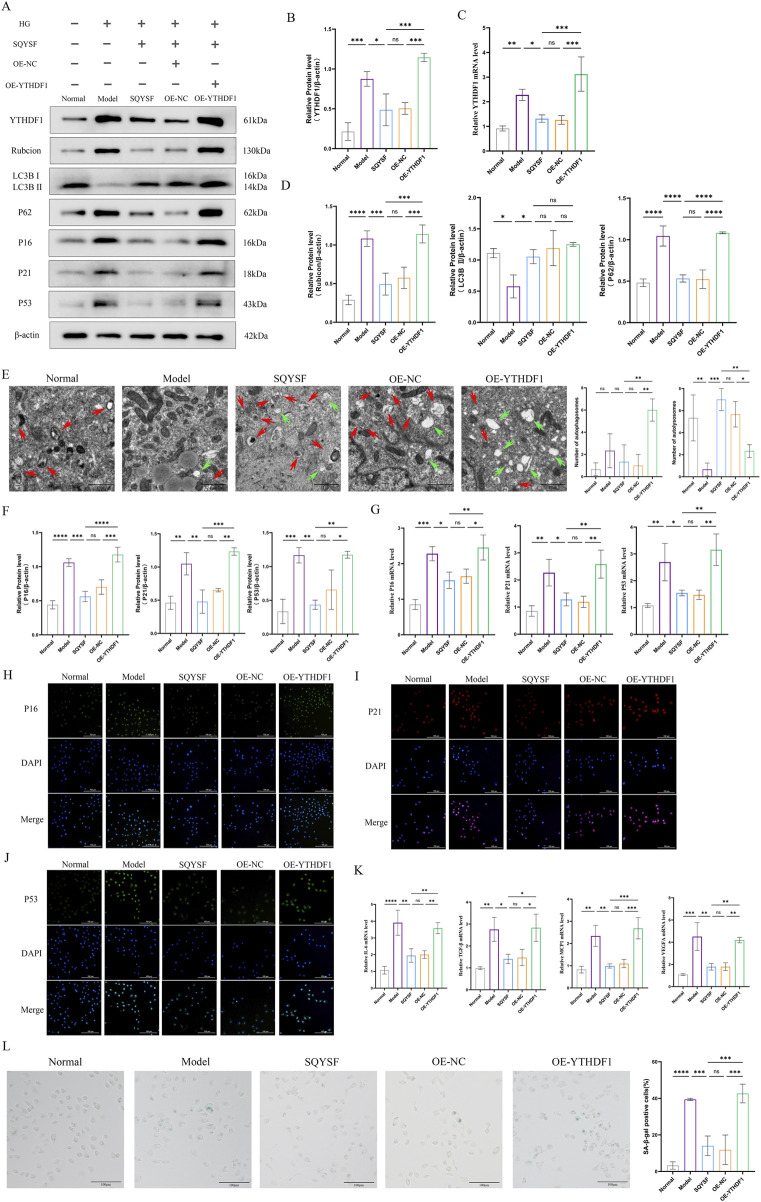
SQYSF promotes autophagy and ameliorates cellular senescence in HK-2 cells by inhibiting the YTHDF1-Rubicon axis. Original bands **(A)**; Quantitative analysis of YTHDF1 protein **(B)**; YTHDF1 mRNA expression levels **(C)**; Quantitative analysis of Rubicon, LC3B II, P62 proteins **(D)**; The number of autophagosome (green arrows) and autolysosome (red arrows) **(E)**; Quantification analysis of P16, P21, and P53 proteins **(F)**; P16, P21, and P53 mRNA expression levels **(G)**; Fluorescence intensity of P16, P21, P53 **(H–J)**; IL-6, TGF-β, MCP1, and VEGFA mRNA expression levels **(K)**; SA-β-gal staining positive cells (L). All data are expressed as means ± SD (n = 3). *P < 0.05, **P < 0.01, ***P < 0.001 and ****P < 0.0001.

To investigate whether SQYSF promotes autophagy to ameliorate cellular senescence through YTHDF1, we constructed a HK-2 cell line with YTHDF1 overexpression and examined whether YTHDF1 overexpression could block the autophagy-promoting and senescence-ameliorating effects of SQYSF-containing serum. The results showed that overexpression of YTHDF1 reversed the autophagy-promoting effect of SQYSF. Western blot showed that overexpression of YTHDF1 increased the expression level of P62 and Rubicon proteins compared to the SQYSF group (Figure 8D). Transmission electron microscopy revealed that overexpression of YTHDF1 led to a reduction in the number of autolysosomes compared to the SQYSF group (Figure 8E). Finally, overexpression of YTHDF1 also inhibited the ability of SQYSF to ameliorate the senescence of HK-2 cells, as confirmed by Western blot, RT-qPCR, and SA-β-gal staining. Compared to the SQYSF group, overexpression of YTHDF1 significantly increased the expression levels of P16, P21, and P53 in HK-2 cells (Figures 8F–J). Furthermore, the mRNA expression levels of IL-6, TGF-β, MCP-1, and VEGFA were elevated (Figure 8K), and SA-β-gal activity was markedly enhanced (Figure 8L). These results indicate that YTHDF1 is a key target of SQYSF in promoting autophagy and ameliorating cellular senescence in high glucose-induced HK-2 cells.

## 4 Discussion

Hyperglycemia is a key driver of cellular senescence and contributes to the progression of DKD (Liang et al., 2022). Recent studies have highlighted the accumulation of senescent cells in DKD, suggesting that targeting the reduction of senescent cells could offer a potentially effective therapeutic approach for DKD (Gao et al., 2022). Some studys show that kidneys of STZ-induced DKD model mice or db/db mice exhibit signs of cellular senescence and damage (Kim MN. et al., 2021; Fang et al., 2021), and similarly, HK-2 cells show overexpression of senescence-related markers under high glucose conditions (Fang et al., 2023). Several commonly used drugs in the clinical management of DKD, such as dapagliflozin and metformin, have been shown to mitigate renal cellular senescence and reduce the expression of senescence markers in DKD (Kim MN. et al., 2021; Liang et al., 2022). Our research revealed that SQYSF not only reduced blood glucose and improved renal function and pathological tissue damage, but also alleviated renal cell senescence and inflammatory state in mice with DKD. It has been reported that typical glomerular structural damage occurs in about 1% of patients with diabetic microalbuminuria, while approximately one-third of patients exhibit little to no glomerular damage but significant tubular epithelial cell injury (Chen et al., 2020). Therefore, it is crucial to investigate the correlation between renal tubular epithelial cells and the deterioration of renal function by using HK-2 cells. *In vitro*, we obtained the similar results as the *in vivo* experiments by using high glucose-intervened HK-2 cells and SQYSF-containing serum as a tested drug.

Autophagy is not only a key mechanism of action in the progression of DKD, but also an important means of regulating cellular senescence (Han et al., 2023; Kang and Elledge, 2016). In our study, we found that SQYSF was able to restore autophagy in DKD kidney in both *in vivo* and *in vitro* experiments, and reverse validation experiments *in vitro* showed that SQYSF could improve HK-2 cellular senescence under high glucose stress by promoting autophagy. Rubicon is known to be located in lysosomes and is thought to be a negative regulator associated with autophagy by binding to class III phosphatidylinositol-3 kinase complexes and aberrantly inhibiting autophagosome-lysosome fusion (Peng et al., 2022). Knockdown of Rubicon using siRNA in podocytes prevented high glucose-induced inhibition of autophagy, suggesting a negative correlation between Rubicon and autophagic fluxes (Li et al., 2021). The expression level of Rubicon can not only be used to monitor the progression of DKD, but its inhibition of autophagic activity has also become a marker of senescence. Therefore, we examined the expression level of Rubicon by RT-qPCR and Western blot, and our results indicated that SQYSF was capable of inhibiting the expression level of Rubicon mRNA and protein. However,the precise molecular mechanisms underlying the modulation of Rubicon expression by SQYSF remain to be elucidated.

We predicted from some studies and databases that Rubicon may undergo m6A modification during DKD progression. M6A methylation modification is a dynamic and reversible cotranscriptional response that plays a key role in the post-transcriptional regulation of gene expression (Jiang et al., 2021). It has been linked to various factors related to the DKD pathogenesis, including inflammatory response, lipid metabolism, cell cycle, oxidative stress, and autophagy (Huang et al., 2024; Lu et al., 2021). In the kidneys of mice with both type 1 and type 2 diabetes, elevated levels of m6A modifications have been reported (Jiang et al., 2022). Moreover, m6A modifications have been implicated in the senescence of cells or tissues associated with the progression of diseases such as endometriosis (Wang X. et al., 2023), osteoarthritis (Chen et al., 2022), and pulmonary fibrosis (Zhang C. et al., 2024). Our findings demonstrated that m6A modification level of Rubicon was increased in the kidneys of DKD mice, but SQYSF failed to reduce the m6A modification level of Rubicon. Therefore, we speculated that SQYSF might regulate the expression level of Rubicon through the Reader molecule, which is due to the fact that the Reader molecule can recognize the m6A site, but not change the m6A modification level (Wang et al., 2015). Then we detected the expression changes of molecules related to m6A modification by RT-qPCR, and the results showed that SQYSF was capable of inhibiting the expression of YTHDF1 mRNA both *in vitro* and *in vivo*. Therefore, the Reader molecule-YTHDF1 may be the key molecule in the modulation of the changes of Rubicon expression by SQYSF. Meanwhile Western blot, RT-qPCR, and immunohistochemistry also revealed that SQYSF was able to inhibit the expression level of YTHDF1, and the middle and high doses of SQYSF were better than the low dose in kidney of db/db mice. Then, to further investigate this, we performed molecular docking between the chemical components of SQYSF and YTHDF1. The docking results indicated that the binding affinity of the compounds to YTHDF1 was less than −1.2 kcal/mol. Additionally, the CETSA showed that the SQYSF-containing serum was able to improve the thermal stability of YTHDF1 protein, making it more resistant to heat treatment. These results suggest that SQYSF has the ability to bind and inhibit YTHDF1.

YTHDF1 can be capable of recognizing m6A modification sites, regulating RNA degradation, stability as well as protein translation, but does not directly affect the m6A modification level of RNA (Wang et al., 2015). It was found that YTHDF1 inhibits autophagy and promotes cell apoptosis by enhancing the stability of Cdc25A mRNA (Liu et al., 2024). To further explore whether YTHDF1 regulates Rubicon expression in the kidney and its underlying mechanism, we used lentivirus to overexpress YTHDF1 in HK-2 cells. Our results demonstrated that YTHDF1 could promote the stability of Rubicon mRNA and increase mRNA and protein expression levels of Rubicon. Although the above results indirectly proved the biological function played by YTHDF1 as a Reader molecule, there is a lack of direct evidence whether YTHDF1 binds to the m6A modification site on Rubicon, and in the future, experiments such as RIP-qPCR, CLIP-seq, or constructing m6A site mutants of Rubicon mRNA can be used to verify the specific binding of YTHDF1 to the Rubicon mRNA and to localise its m6A modification site. Rubicon mRNA specific binding and locate its m6A modification site. In addition, these experiments were conducted *in vitro*, and further studies involving YTHDF1 knockout or overexpression in db/db mice, as well as in normal mice, are needed to better understand its molecular mechanisms in different physiological and pathological contexts. Research has shown that YTHDF1 is implicated in the progression of renal fibrosis and is highly expressed in human fibrotic kidneys (Xing et al., 2022). In addition, YTHDF1 was also upregulated in diabetic cataract samples (Cai et al., 2023). Our results revealed that YTHDF1 is highly expressed in the kidney of db/db mice and in HK-2 cells under high glucose stress. The ability of SQYSF to reduce the expression level of YTHDF1 may be a key point to ameliorate the cellular senescence in DKD. Finally, by overexpressing YTHDF1 *in vitro*, we found that the beneficial effects of SQYSF on Rubicon, autophagy, and cellular senescence were reversed in an *in vitro* model of DKD.

**FIGURE 9:**
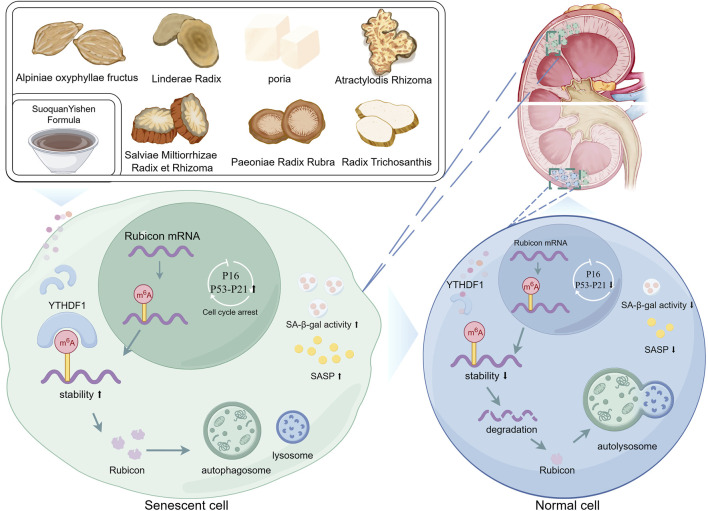
Graphical abstract (by Figdraw).

Our study links the traditional Chinese medicine theory of “warming the spleen and tonifying the kidneys or clearing up Zhenjia” with the modern scientific concept of “cellular senescence,” and demonstrates that SQYSF can improve cellular senescence in DKD kidney. Through an in-depth investigation of the molecular mechanisms of the drug, we found that SQYSF could enhance autophagy, thereby alleviating cellular senescence. Furthermore, we report for the first time that YTHDF1 can increase the stability of Rubicon mRNA and promote protein translation, which may represent a key pathway in the progression of DKD. SQYSF was found to target and regulate YTHDF1, thereby modulating Rubicon-mediated autophagy and exerting therapeutic effects on cellular senescence in DKD kidney. In summary, targeting and regulating the YTHDF1-Rubicon axis-mediated cellular autophagy, thereby exerting a therapeutic effect on cellular senescence in DKD kidneys is an important pathway for SQYSF to ameliorate DKD (Figure 9).

## 5 Conclusion

The results suggest that YTHDF1 and autophagy are key factors in the ability of SQYSF to improve renal cellular senescence in DKD. The therapeutic effects of SQYSF in ameliorating renal cellular senescence in DKD are mediated through its targeted binding and regulation of YTHDF1, which influences the stability of Rubicon mRNA and its protein translation, thereby promoting autophagy. These findings not only reveal the potential molecular mechanisms underlying SQYSF’s effects in treating DKD but also suggest that targeting YTHDF1 may provide a novel approach for the prevention and treatment of DKD.

## Data Availability

The original contributions presented in the study are included in the article/Supplementary Material, further inquiries can be directed to the corresponding authors.
