# Supplementary figures and images for: SuoquanYishen formula improves renal cellular senescence by inhibiting YTHDF1-Rubicon axis to promote autophagy in diabetic kidney disease
Source: Front Pharmacol. 2025 Apr 30;16:1543277. doi: 10.3389/fphar.2025.1543277 (PMC12075247; doi:10.3389/fphar.2025.1543277)

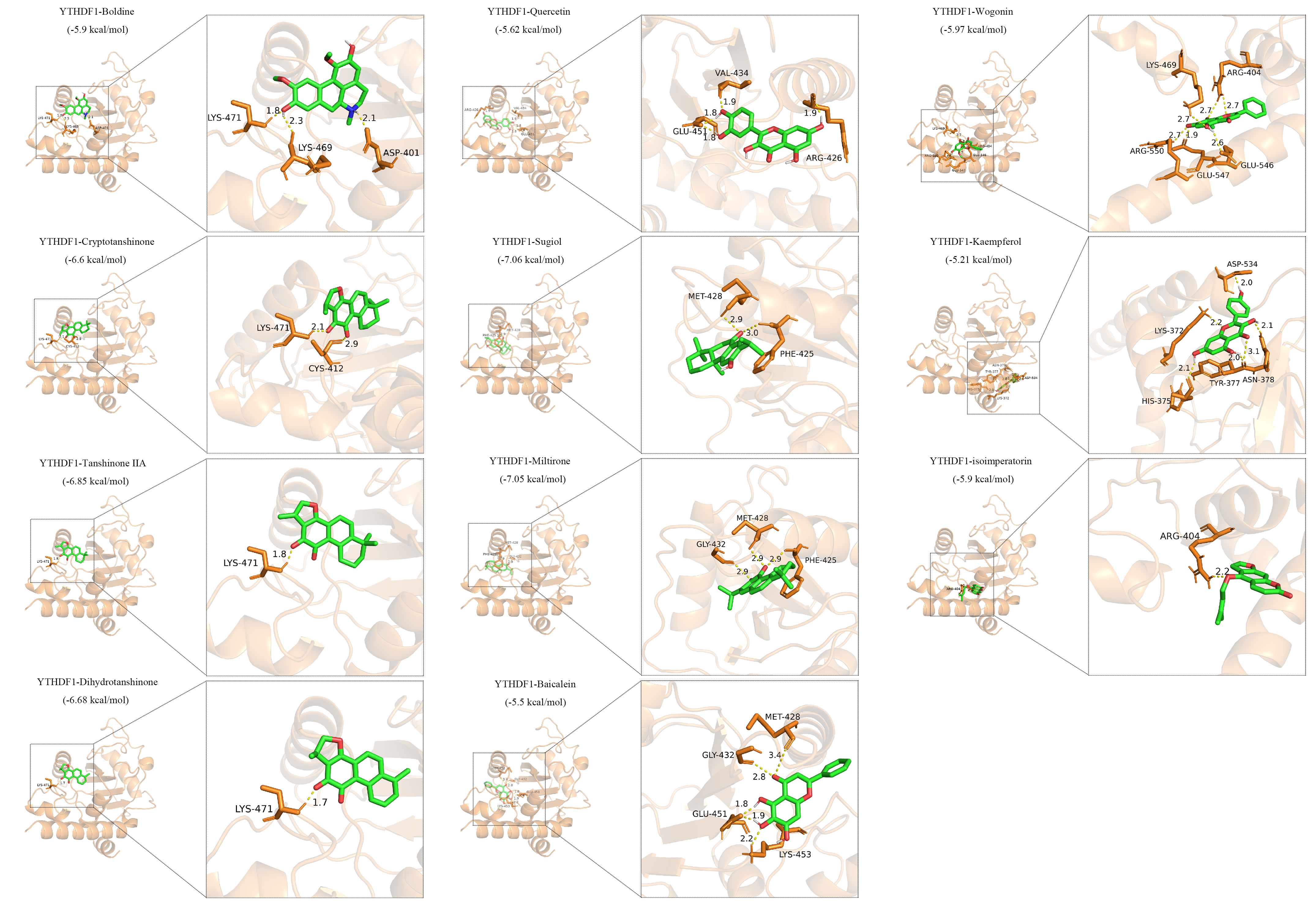

Supplement: Supplementary file 2 [file Image3.jpeg]

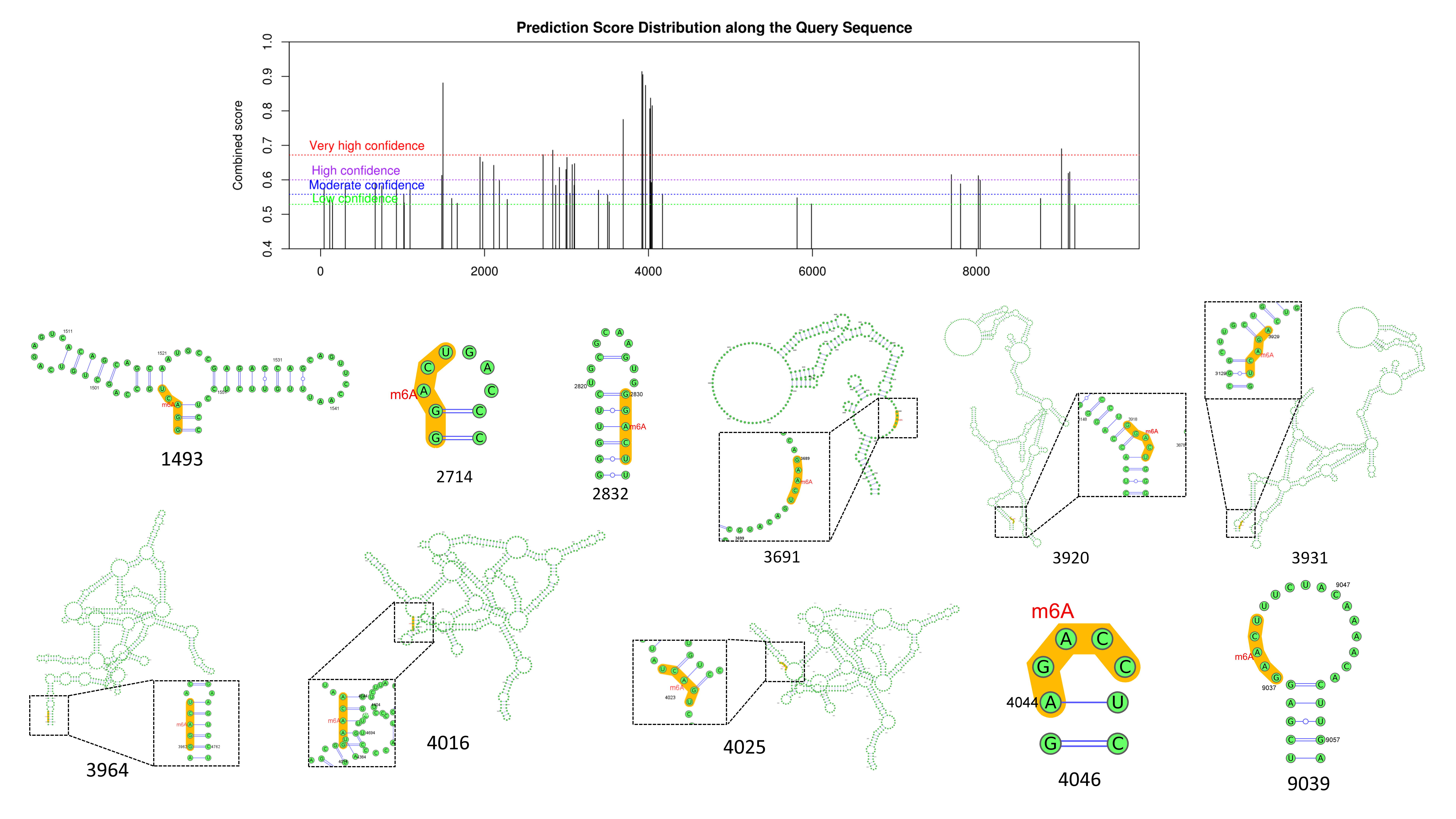

Supplement: Supplementary file 4 [file Image1.jpeg]

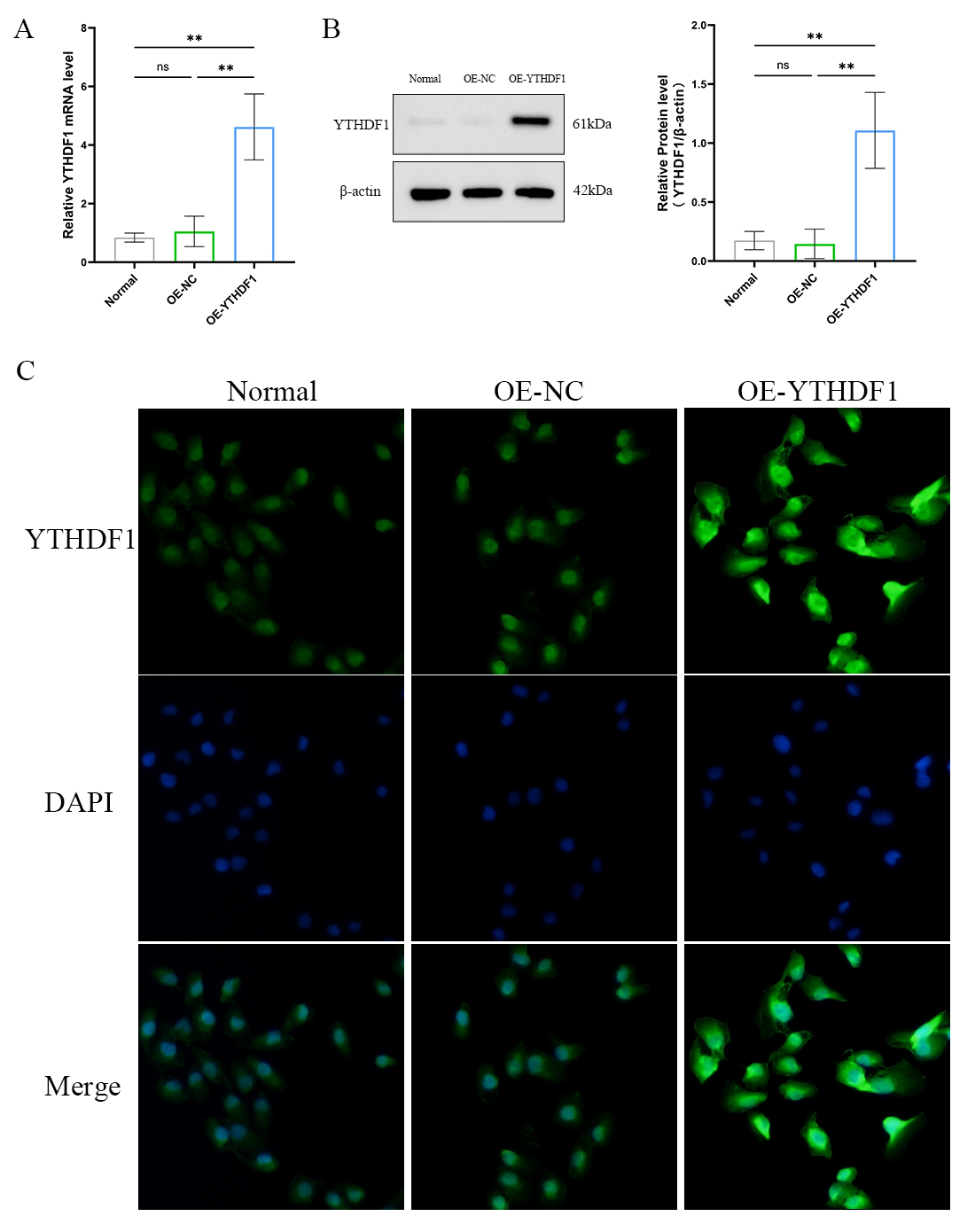

Supplement: Supplementary file 5 [file Image2.jpeg]
